# Sociodemographic Attributes and Prevalence of Arrest With Possession of Substances in Incarcerated Population in the United States

**DOI:** 10.7759/cureus.22379

**Published:** 2022-02-19

**Authors:** Manpreet Gill, Afrina Zaman, Jisha Kallikkadan, Oluwatoyin Oladeji, Samuel Adeyemo, Stanley Nkemjika, Terence Tumenta, Stephanie Madubuike, Gurraj Singh, Olalekan Olaolu, Tolu Olupona

**Affiliations:** 1 Psychiatry, Interfaith Medical Center, New York, USA; 2 Population Health Sciences, Georgia State University, School of Public Health, Atlanta, USA; 3 Psychiatry and Behavioral Sciences, Interfaith Medical Center, New York, USA; 4 Psychiatry, American University of Antigua, Coolidge, ATG; 5 Psychiatry, Sri Guru Ram Das Institute of Medical Sciences and Research, Amritsar, IND

**Keywords:** correctional psychiatry, sociodemographic attributes, substance use, possession of substance, inmates

## Abstract

Background and objectives

In recent years, there has been an increase in the US imprisonment rate. A substantial percentage of those incarcerations are for drug-related offenses. The authors investigated the relationship between the pattern of substance use and drug-related offenses across a broad spectrum of various sociodemographic attributes of the incarcerated population in the United States.

Methods

Cross-sectional data from the 2016 Survey of Prison Inmates conducted by the Bureau of Judicial Statistics were extracted with inmates who reported possession of a drug at the time of arrest as a primary outcome of interest. Using SAS 9.4 statistical software (SAS Institute Inc., Cary, USA), the authors used multivariate analyses to determine the odds ratios between various sociodemographic attributes of the inmates and possession of substance at the time of the arrest. Logistic regression analysis for age groups in relation to substance possession at the time of arrest is presented in the form of an adjusted odds ratio and their respective confidence interval at p ≤0.5.

Results

Out of the total 23,798 inmates who reported possession of a drug at the time of arrest, 34.07% were Non-Hispanic Whites, and 31.5% were within the age group of 25-34 years. Only 59.47% of inmates were employed 30 days before the arrest, and 58.02% had less than a high school education. Different patterns of drug use were linked with different types of drugs found in their possession at the time of the arrest. Possession of cannabis at the time of arrest was highest in the age group 18-24 years compared to other age groups (odds ratio: 1.362; 95% CI: 1.159 - 1.602). Inmates with a history of stimulant or hypnotic use were more likely to have another psychoactive substance during a time of the arrest. Only 8.46% of inmates had psychiatric and psychological treatment as part of their sentence.

Conclusions

A large proportion of incarcerations in the US is because of drug-related offenses, with most of the burden on the younger age group. Inmates should receive psychiatric and psychological treatments for substance use as part of their sentencing while in prison and after release as a form of targeted intervention for this vulnerable group.

## Introduction

The United States (U.S.) has led the world in imprisonment rates, going back to the last quarter of the twentieth century. The imprisonment rate in the U.S. in 2020 was near 0.7% of the total U.S. population [1], equivalent to 20% of the world's total incarcerated population [2], despite representing only 4.25% of the world population [3]. More than 2.2 million adults are incarcerated in U.S. federal prisons, state prisons, or local jails [1]. Since the enactment of the Comprehensive Drug Abuse Prevention and Control Act of 1970 and the global campaign "War on Drugs" initiative during President Nixon's tenure, that figure has inflated due to the high rates of drug-affiliated felonies and related criminal behavior, along with minimal to no change in drug policy and criminal sentencing [4]. 

The overwhelming population of incarcerated prison inmates is correlated to drug-related offenses, showing a significant association and high prevalence between substance use and criminality [5]. Literature showed that 32% of those incarcerated were under the influence of a substance when committing a crime, and 56% of the state prison inmates had taken at least one illicit substance in the month before their offense [3]. Amongst the inmate population, particularly in females, substance abuse was observed, with the highest prevalence, primarily with alcohol (30.2%) and cocaine dependence (30.1%). Other substances included: stimulant dependence (24.1%); marijuana dependence (15.6%); heroin dependence (9.6%) [6]. In comparison, in 2018, approximately 20.3 million people aged 12 or older were reported to have a substance use disorder (SUD), with those in jail (53%), in-state prisons (56%), and in federal prisons (50%) meeting the criteria for SUD [7]. Those seen with SUD had, on average multiple incarcerations (78.8%), a higher rate of juvenile convictions (60.2%), greater violent behavioral outbursts during detentions (29.8%), and a history of one or more suicide attempts (20.8%) [8]. A history of repeated substance use, along with psychiatric hospital admissions, poor living conditions, gender, early age of first use, plus antisocial personality disorder are all distinguishing predictors for future, repeated offenses [9]. 

Adolescents have also shown a positive association with drug-related criminal behavior. This undeniable link between substance use and delinquency often results in gang affiliations, drug trafficking, prostitution, and youth homicides [10]. This link is predominant in male adolescents more than females, with severity and frequency of various substance use at different rates and with different consequences [11]. In 2019, law enforcement agencies in the U.S. made an estimated 697,000 arrests under the age of 18, with 17% of the total offenses relating to substance use: drug abuse violations (81,320); liquor laws (26,650); driving under the influence (5,570); and drunkenness (3,470) [12]. Based on data collected by the U.S. Office of Juvenile Justice and Delinquency Prevention from urinalysis results performed on youth detainees, 85.4% had used some kind of illicit substance in the past six months, and 94% had used an illicit substance at some point in their life [13]. Evidence in literature has suggested that over two-thirds of incarcerated adolescents have at least one SUD [14]. If the onset of substance use precedes the age of 16 years, there is a fourfold increased risk (0.35 vs. 0.09, p=0.044) of incarceration during adulthood for substance use-related offenses [15]. Research also infers that those criminal arrests increase the risk of substance use and exacerbate existing substance use over time, which is associated with greater criminal behavior in young adulthood and early midlife. Subsequently, this consequentially results in greater criminal arrests in later midlife [16]. 

Untreated substance use disorders amongst the newly released incarcerated population can culminate in relapse. They are further subjected to criminal-like behavior, leading to probation or parole violations and a high chance of re-incarceration [17]. Studies have also reported that those newly released from jail or prison showed an increased risk associated with drug overdose-related death [18,19] or were highly susceptible to transmitting viral infections such as hepatitis and HIV through needle injections [20]. Thus, it demonstrates a public health emergency and a health policy concern. Hence, the need for expanding mental health services to contend with the high number of drug-dependent prison inmates and newly released offenders with substance-related addictions. 

In this study, we aim to investigate the relationship between substance use linked with criminal behavior amongst the incarcerated population across a broad spectrum of various sociodemographic attributes, including but not limited to age, sex, race, marital status, education, and employment status. Researching the potential trajectory of substance use history and criminality can aid in the development of targeted interventions for the most vulnerable groups who are at the highest risk. In addition, this study will be useful for educational and policy purposes for public program initiatives and support groups about the awareness and implications of long-term physical, mental and social consequences of chronic substance use. 

## Materials and methods

Study population

Our study utilized data from the 2016 Survey of Prison Inmates (SPI) conducted by the Bureau of Justice Statistics (BJS), which provides a nationally representative of persons incarcerated in state or federal correctional facilities within the United States during the year 2016. The SPI contains information on all males and females aged 18 years and above incarcerated in a government facility. The SPI included both confinement and community-based facilities, except facilities operated by or holding exclusively for the U.S. military, Immigration and Customs Enforcement, the U.S. Marshals Service, and correctional authorities in Indian country. The dataset is cross-sectional and provides information on the prisoners' characteristics such as current offenses, incidence characteristics, judgment sentence characteristics, criminal history, firearm possession, substance use attributes, sociodemographic attributes, mental health services, treatment, and facility programs rules violations, etc. The dataset consisted of 2,001 unique prisons housing 1,502,671 prisoners, with 1,400,363 representing male prisoners and 102,308 representing female prisoners.

Data collection and sampling

SPI data were collected via face-to-face interviews with inmates utilizing computer-assisted personal interviewing (CAPI), which was voluntary. Based on our present study, we included all inmates who responded to the questionnaire on possession of substance of abuse at the time of the arrest. The final sample derived from the data based on our interest is 23,798 inmates housed in all government facilities. SPI utilized a two-stage sample design, with the first stage consisting of a random sample of prisons. The sampling frame for the prisons was based on the updated 2012 census conducted by the department of justice in the correctional facilities. It was stratified by the particular sex housed in the facility, jurisdiction (state or federal), and self-representing states (i.e., states that housing >100,000 inmates as of December 31, 2013). Facilities housing biological sexes were split into two sampling units based on physical sex orientation and placed in the appropriate prisoner sex stratum. The second stage of the design consisted of a random sample of prisoners held in the selected prisons. Within each stratum, SPI was an approximate equal probability selection method (EPSEM) design. This process ensured that a nonresponse was constant across facilities with a constant number of prisoners per facility selected. Hence, the procedure provided self-weighing, giving each respondent within a stratum (regardless of facility size) the same probability of selection. This process minimized the variance of national estimates, which represents a novelty in the 2016 SPI. 

Ethical consideration

We utilized a secondary de-identified dataset that is available to the public. The Institutional Review Board at the Interfaith Medical Center, Brooklyn, NY, waived the review process.

Measures of variables

The outcome variable is a binary variable "POSSESSION OF A DRUG" at the time of the arrest. The response is either "1=yes" or "2=no". This variable was further re-categorized into drug types which include cannabis, cocaine, crack, ecstasy, methamphetamines, hallucinogens, prescription opioids, heroin, phencyclidine, etc. Our independent or predictor variables are mainly the inmates' sociodemographic attributes and confounding variables identified via literature. These independent variables include age (ordinal group), sex, marital status, educational attainment, employment status, and firearm possession at the time of the arrest.

Statistical analysis

We analyzed all included variables. In the descriptive statistics, the frequency distribution (prevalence) was reported to describe the characteristics of the population. We also ran a bivariate descriptive of the outcome of interest with the type of substance used in the past 30 days before the arrest. Bivariate analysis was performed to examine the crude odds ratio (OR) between sociodemographic attributes and possession of substance at the time of the arrest. Subsequent multivariate analysis was conducted, controlling for confounding covariates. We conducted separate ordinal logistic regression analyses for age groups in relation to substance possession at the time of the arrest. Our results were presented in the form of adjusted odds ratio (AOR) and their respective 95% confidence intervals (CI) at a p≤0.05. All analyses were done using SAS 9.4 statistical software (SAS Institute Inc., Cary, USA).

## Results

Sociodemographic attributes of the population

The age distribution of our study population showed that the age groups 25-34 years and 35-44 years had the greatest distribution with 31.50% and 27.98%, respectively. Other notable age groups are the 55-64 years and 65+ years which had the lowest frequencies as 8.92% and 2.61%, respectively. With regards to racial distribution, non-Hispanic White (NHW) inmates have the largest frequency (34.07%), followed by non-Hispanic Blacks (NHB) with 30.12%. Our population sample was predominantly male, with a prevalence of 74.34%. For marital status, most of the inmates were either never have been married (55.66%) or have been divorced (19.82%). The educational level of inmates showed that the prevalence reduced based on a higher level of educational attainment, as a total of 13,808 (58.02%), 5,524 (23.21%), 3,193 (13.42%), and 1,273 (5.35%) represents <high school, high school, some college, and >college level, respectively. Out of the inmates, 18.23% were in possession of firearms at the time of the arrest. Substance use treatment program included in their sentence had 20.17%, 8.46% were receiving a form of psychiatric treatment, and 84.11% were in possession of psychoactive substance at the time of the arrest. See Table 1 for more sociodemographic attributes. In terms of sociodemographic attributes by type of psychoactive substance in possession at the time of arrest, see Table 2.

**Table 1:** Socio-demographic descriptive of 2016 inmates (n=23,798) NH - Non-Hispanic; PCP - phencyclidine

Characteristics	N	%	p-value
Current age			<0.0001
18-24	2256	9.48	
25-34	7496	31.5	
35-44	6659	27.98	
45-54	4644	19.51	
55-64	2122	8.92	
65 or older	621	2.61	
Race/Hispanic origin			<0.0001
White (NH)	8107	34.07	
Black (NH)	7167	30.12	
Hispanic	5073	21.32	
American Indian/Alaska Native (NH)	339	1.42	
Asian/Native Hawaiian/other Pacific Islander (NH)	209	0.88	
2+ races (NH)	2605	10.95	
Other (NH)	7	0.03	
Uncategorized – missing	291	1.22	
Biological sex			<0.0001
Male	17692	74.34	
Female	6106	25.66	
Marital status			<0.0001
Married	3723	15.64	
Widowed	742	3.12	
Separated	1371	5.76	
Divorced	4716	19.82	
Never married	13246	55.66	
Educational attainment (4 levels) - highest year of education completed prior to prison			<0.0001
Less than high school	13808	58.02	
High school graduate	5524	23.21	
Some college	3193	13.42	
College degree or more	1273	5.35	
Possessed firearm at time of offense			<0.0001
Yes	4291	18.23	
No	19253	81.77	
Does sentence include drug/alcohol treatment program: original			<0.0001
Yes	4730	20.17	
No	18715	79.83	
Does sentence include psychiatric/psychological counseling/treatment: original			<0.0001
Yes	1983	8.46	
No	21462	91.54	
Any offenses a "possession" of drug			<0.0001
Yes	4087	84.11	
No	772	15.89	
Lived in family-owned house/apartment/etc. 30 days before arrest			<0.0001
Yes	19987	84	
No	3807	16	
Any jobs worked 30 days before arrest			<0.0001
Yes	13927	59.47	
No	9490	40.53	
Used marijuana/hashish 30 days before arrest			
Yes	10963	54.93	
No	8996	45.07	
Used any form of cocaine 30 days before arrest			
Yes	4053	33.92	
No	7896	66.08	
Used crack 30 days before arrest			
Yes	1864	31.97	
No	3966	68.03	
Used heroin 30 days before arrest			
Yes	2103	44.19	
No	2656	55.81	
Used PCP 30 days before arrest			
Yes	426	16.19	
No	2206	83.81	
Used ecstasy or Molly 30 days before arrest			
Yes	1625	23.3	
No	5348	76.7	
Used other hallucinogens 30 days before arrest			
Yes	448	7.39	
No	5617	92.61	
Used methamphetamine 30 days before arrest			
Yes	3874	57.19	
No	2900	42.81	
Used inhalants 30 days before arrest			
Yes	152	6.06	
No	2356	93.94	
Used prescription drugs not prescribed 30 days before arrest			
Yes	4163	51.01	
No	3998	48.99	
Used other drugs just for kicks 30 days before arrest			
Yes	323	22.81	
No	1093	77.19	

**Table 2:** Sociodemographic descriptive of inmates in possession of drugs prior to arrest among 2016 national data CBD - cannabis/cannabinoids; COC - cocaine; CRK - crack; OPD - opioid; PCP - phencyclidine; MDMA - ecstasy; AMPH - amphetamines; HAL -hallucinogens; Rx - prescription opioids; other - other psychoactive substance

Characteristics	Possession of drugs										p-value
	CBD	COC	CRK	OPD	PCP	MDMA	HAL	AMPH	Rx	OTHER	
Current age											<0.0001
18-24	10.12	9.63	3.66	3.87	10.09	0	13.04	5.79	9.18	11.58	
25-34	31.01	37.33	27.61	30.41	40	20	52.17	35.7	36.71	26.32	
35-44	26.63	28.72	39.49	41.24	29.01	60	30.43	34.3	27.05	28.42	
45-54	19.78	17.06	20.66	20.1	13.33	10	4.35	18.43	19.32	20	
55-64	9.54	6.25	6.95	3.61	6.49	10	0	4.88	6.28	13.68	
65 or older	2.92	1.01	1.65	0.77	1.08	0	0	0.91	1.45	0	
Race/Hispanic origin											<0.0001
White	34.66	25.84	10.33	7.99	29.01	0	21.74	53.22	69.57	57.89	
Black	30.49	26.86	47.35	66.49	30.63	80	30.43	3.06	8.21	9.47	
Hispanic	20.01	32.6	33.27	10.57	28.47	0	26.09	28.76	8.21	20	
American Indian/Alaska Native	1.5	1.52	0.37	0.52	0.18	0	0	2.07	1.93	0	
Asian/Native Hawaiian/other Pacific Islander	0.87	2.03	0.64	0	0.54	0	17.39	0.99	0.48	0	
2+ Races	11.22	9.8	6.49	11.6	9.91	20	4.35	11.32	11.11	12.63	
Other	0.04	0	0	0	0	0	0	0	0	0	
Uncategorized – missing	1.22	1.35	1.55	2.84	1.26	0	0	0.58	0.48	0	
Sex for analysis											<0.0001
Male	75.54	79.39	83	76.8	68.11	100	65.22	55.12	40.1	41.05	
Female	24.46	20.61	17	23.2	31.89	0	34.78	44.88	59.9	58.95	
Marital status											<0.0001
Married	15.15	17.74	19.56	14.69	15.32	40	13.04	18.51	16.43	25.26	
Widowed	3.28	1.86	2.29	1.8	1.98	0	0	2.73	3.38	5.26	
Separated	5.5	7.09	5.76	3.87	5.59	0	4.35	9.5	9.18	6.32	
Divorced	20.19	16.55	14.44	11.6	12.61	10	13.04	25.95	22.22	18.95	
Never married	55.88	56.76	57.95	68.04	64.5	50	69.57	43.31	48.79	44.21	
Educational attainment (4 levels) - highest year of education completed prior to prison)											<0.0001
Less than high school	57.78	59.97	61.33	72.68	56.04	60	47.83	56.45	51.69	45.26	
High school graduate	23.04	24.16	23.22	18.56	23.24	40	21.74	25.79	28.02	27.37	
Some college	13.45	13.34	11.15	7.47	16.76	0	26.09	14.46	15.46	18.95	
College degree or more	5.73	2.53	4.3	1.29	3.96	0	4.35	3.31	4.83	8.42	
Possessed firearm at time of offense											<0.0001
Yes	20.02	12.84	10.15	11.6	7.21	0	13.04	10.91	1.45	3.16	
No	79.98	87.16	89.85	88.4	92.79	100	86.96	89.09	98.55	96.84	
Does sentence include drug/alcohol treatment program											<0.0001
Yes	17.27	35.7	28.7	33.07	33.7	20	34.78	37.55	32.37	33.68	
No	82.73	64.3	71.3	66.93	66.3	80	65.22	62.45	67.63	66.32	
Does sentence include psychiatric/psychological counseling/treatment											<0.0001
Yes	8.7	5.08	4.57	6.72	7.1	0	8.7	9.76	13.04	13.68	
No	91.3	94.92	95.43	93.28	92.9	100	91.3	90.24	86.96	86.32	
Any offenses a "possession" of drug											<0.0001
Yes	1.46	99.16	98.26	96.65	97.3	90	100	97.6	94.69	95.79	
No	98.54	0.84	1.74	3.35	2.7	10	0	2.4	5.31	4.21	
Lived in family-owned house/apartment/etc. 30 days before arrest											0.0058
Yes	83.76	86.15	88.48	84.54	83.24	90	91.3	83.14	84.06	78.95	
No	16.24	13.85	11.52	15.46	16.76	10	8.7	16.86	15.94	21.05	
Any jobs worked 30 days before arrest											<0.0001
Yes	60.95	59.97	58.46	38.54	45.36	80	63.64	50.84	53.23	52.13	
No	39.05	40.03	41.54	61.46	54.64	20	36.36	49.16	46.77	47.87	

Relationship between prior substance use and possession of psychoactive substances on arrest

Inmates who have ever used crack were mainly arrested in possession of heroin (61.84%), phencyclidine (58.68%), and prescription opioids (55.00%). Similar inmates were less likely to be arrested in possession of cocaine (38.06%) and crack (36.40%). For inmates who have ever used heroin, those in possession of prescription opioids had the highest prevalence of 35.75%, while none were in possession of methamphetamine. Possession of amphetamines had the highest prevalence for those who have ever used phencyclidine (PCP), with a frequency of 40%. Alternatively, for those who have used ecstasy in the past, possession of hallucinogens had the highest prevalence of 82.61%, and cannabis had the lowest prevalence of 27.58%. Inmates who have ever used hallucinogens had the highest prevalence of possessing amphetamines at the time of arrest with the prevalence of 46.60%, while those with past use of Methamphetamines had the highest prevalence of use for the same substance (84.33%). Similarly, the distribution shows that inmates with past use of inhalants (type of hallucinogen) had the highest prevalence of possessing a hallucinogen at the time of arrest (21.74%). Inmates in possession of crack had the least prevalence among those who have ever used inhalants, with a frequency distribution of 3.95%. See Table 3 for more details.

**Table 3:** Relationship between prior substance use and getting arrested with possession of drugs CBD - cannabis/cannabinoids; COC - cocaine; CRK - crack; OPD - opioid; PCP - phencyclidine; MDMA - ecstasy; AMPH - amphetamines; HAL -hallucinogens; Rx - prescription opioids; other - other psychoactive substance

Characteristics	Possession of drugs										p-value
	CBD	COC	CRK	OPD	PCP	MDMA	HAL	AMPH	Rx	OTHER	
Have you ever used crack?											<0.0001
Yes	48.92	38.06	36.4	61.84	58.68	0	41.67	51.89	55	50.77	
No	51.08	61.94	63.6	38.16	41.32	100	58.33	48.11	45	49.23	
Have you ever used heroin?											<0.0001
Yes	19.28	16.5	10.84	11.34	51.99	0	17.39	27.78	35.75		
No	80.72	83.5	89.16	88.66	48.01	100	82.61	72.22	64.25		
Have you ever used PCP?											0.0018
Yes	11.06	9.01	9.18	13.92	12.82	40	21.74	12.69	9.66	9.47	
No	88.94	90.99	90.82	86.08	87.18	60	78.26	87.31	90.34	90.53	
Have you ever used ecstasy or Molly?											<0.0001
Yes	27.58	35.37	29.48	34.79	44.22	30	82.61	43.53	40.58	41.05	
No	72.42	64.63	70.52	65.21	55.78	70	17.39	56.47	59.42	58.95	
Have you ever used any other type of hallucinogen?											<0.0001
Yes	24.83	26.19	13.96	9.54	32.13	0	39.13	46.6	36.71	42.11	
No	75.17	73.81	86.04	90.46	67.87	100	60.87	53.4	63.29	57.89	
Have you ever used methamphetamine?											<0.0001
Yes	26.28	28.91	11.02	8.25	26.71	0	26.09	84.33	43.48	52.63	
No	73.72	71.09	88.98	91.75	73.29	100	73.91	15.67	56.52	47.37	
Have you ever used inhalants?											<0.0001
Yes	10.61	9.86	3.95	4.9	9.39	10	21.74	17.33	16.43	12.63	
No	89.39	90.14	96.05	95.1	90.61	90	78.26	82.67	83.57	87.37	

Logistic regression estimates for possession of substance at the time of arrest by sociodemographic attribute

In terms of sociodemographic relationship to possession of substance at the time of arrest, age was noted to be statistically significant with possession of cannabis [AOR=1.158 (95% CI: 1.059-1.267; p=0.0015), cocaine [AOR=0.820 (95% CI: 0.765-0.878; p<0.0001)], crack [AOR=0.866 (95% CI: 0.799-0.982; p<0.0001)], heroin [AOR=1.184 (95% CI: 1.079-1.298; p<0.0001)] and ecstasy [AOR=1.921 (95% CI: 1.168-3.161; p<0.05)] adjusting for race, biological sex, marital status, education, possession of firearm, and employment status. Racial predisposition had a statistically significant association with possession of cannabis [AOR=0.944 (95% CI: 0.895-0.995; p=0.0351)], cocaine [AOR=0.948 (95% CI: 0.907-0.990; p=0.0172)], crack [AOR=0.919 (95% CI: 0.864-0.977; p<0.01)], methamphetamines [AOR=1.079 (95% CI: 1.032-1.128; p<0.0001)] and prescription treatment [AOR=1.289 (95% CI: 1.149-1.447; p<0.01)] adjusting for other sociodemographic covariates.

Biological sex as a sociodemographic attribute had a statistical significant relationship at the time of arrest with possession of cannabis [AOR=1.797 (95% CI: 1.447-2.233; p<0.0001)], cocaine [AOR=2.611 (95% CI: 2.183-3.123; p=0.01)], crack [AOR=1.465 (95% CI: 1.138-1.888; p<0.01)], methamphetamines [AOR=0.438 (95% CI: 0.378-0.506; p<0.0001)] and prescription treatment [AOR=0.307 (95% CI: 0.226-0.416; p<0.0001)] adjusting for other sociodemographic attributes. The marital status of the inmates at the time of arrest showed a statistically significant relationship with possession of crack [AOR=0.866 (95% CI: 0.798-0.939; p<0.0001)], heroine [AOR=0.927 (95% CI: 868-0.990; p<0.05)] and methamphetamines [AOR=1.097 (95% CI: 1.048-1.148; p<0.0001)] adjusting for sociodemographic covariates. Additionally, educational attainment level at the time of arrest had statistically significant association with possession of crack [AOR=1.481 (95% CI: 1.266-1.733; p<0.0001)], heroine [AOR=0.853 (95% CI: 0.767-0.949; p<0.0001)] and other uncategorized substances [AOR=0.794 (95% CI: 0.640-0.985; p<0.01)] adjusting for other sociodemographic attributes. The possession of firearms at the time of arrest had a statistically significant association with possession of heroin [AOR=0.615 (95% CI: 0.434-0.868; p<0.0001)], methamphetamines [AOR=1.314 (95% CI: 1.047-1.649; p<0.01)] and prescription treatment [AOR=0.159 (95% CI: 0.050-0.501; p<0.0001)] adjusting for other sociodemographic attributes. Finally, employment status at the time of arrest had a statistically significant association with possession of cannabis [AOR=1.360 (95% CI: 1.133-1.633; p<0.01)], cocaine [AOR=1.250 (95% CI: 1.081-1.446; p<0.0001)] and heroin [AOR=0.704 (95% CI: 0.585-0.847; p<0.0001)] adjusting for other sociodemographic attributes. See Table 4 for more details.

**Table 4:** Odds of possession of substance at the time of arrest by sociodemographic attributes in a logistic regression CBD - cannabis/cannabinoids; COC - cocaine; CRK - crack; OPD - opioid; PCP - phencyclidine; MDMA - ecstasy; AMPH - amphetamines; HAL - hallucinogens; Rx - prescription opioids; other - other psychoactive substance *p<0.05, **p<0.01, ***p<0.001; No estimates for hallucinogens due to low frequencies.

Characteristics	Adjusted odds ratio for possession of drugs [AOR (95% confidence interval)]								
	CBD	COC	CRK	OPD	PCP	MDMA	AMPH	Rx	OTHER
Age	1.158	0.82	0.886	1.184	0.952	1.921	1.042	1.04	0.93
	(1.059-1.267)**	(0.765-0.878)*	(0.799-0.982)*	(1.079-1.298)***	(0.524-1.728)	(1.168-3.161)*	(0.973-1.116)	(0.900-1.201)	(0.761-1.136)
Race	0.944	0.948	0.919	0.987	0.933	0.911	1.079	1.289	1.114
	(0.895-0.995)*	(0.907-0.990)*	(0.864-0.977)**	(0.934-1.042)	(0.642-1.355)	(0.725-1.145)	(1.032-1.128)***	(1.149-1.447)**	(0.969-1.281)
Sex	1.797	2.611	1.465	1.087	-	1.054	0.438	0.307	0.344
	(1.447-2.233)**	(2.183-3.123)*	(1.138-1.888)**	(0.890-1.327)		(0.416-2.667)	(0.378-0.506)**	(0.226-0.416)**	(0.223-0.530)*
Marital status	1.005	0.977	0.866	0.927	1.216	0.912	1.097	1.002	1.123
	(0.947-1.067)	(0.931-1.025)	(0.798-0.939)***	(0.868-0.990)*	(0.844-1.753)	(0.662-1.255)	(1.048-1.148)***	(0.908-1.106)	(0.986-1.280)
Education	0.997	1.021	1.481	0.853	1.391	0.688	1.003	0.945	0.794
	(0.895-1.110)	(0.936-1.113)	(1.266-1.733)**	(0.767-0.949)**	(0.569-3.397)	(0.433-1.092)	(0.924-1.088)	(0.803-1.111)	(0.640-0.985)*
Firearm	1.282	1.011	1.138	0.614	-	1.317	1.314	0.159	0.369
	(0.976-1.684)	(0.797-1.281)	(0.812-1.594)	(0.434-0.868)**		(0.383-4.529)	(1.047-1.649)*	(0.050-0.501)**	(0.115-1.179)
Job	1.36	1.25	0.542	0.704	2.845	1.599	0.999	1.194	1.043
	(1.133-1.633)**	(1.081-1.446)**	(0.435-0.675)	(0.585-0.847)***	(0.596-13.583)	(0.657-3.889)	(0.868-1.150)	(0.890-1.601)	(0.685-1.588)

Association between age at the time of arrest and possession of substance

Following the different ordinal cadre of age groups, we modeled the ordinal logistic regression to assess whether the possession of the different substances at the time of arrest is similar across different age groups. We included the biological sex, marital status, level of education attained at the time of arrest, possession of a firearm, and employment status in the model as probable confounding variables based on the association of these variables with different age groups from previous literature. The odds of possessing cannabis at the time of arrest among inmates of low age group (18-24 years vs. others, or the combined 18-24, 25-34, 35-44, 45-54, 55-64 years vs. >65 years) is about 1.362 times compared to those not in possession of cannabis with the same level of biological sex, marital status, education, possession of firearm and employment status. The 95% confidence interval of the odds ratio is OR: 1.362; 95% CI: 1.159-1.602; p=0.0002. Similarly, the odds of possessing cocaine or crack at the time of arrest among inmates of low age group (18-24 years vs. others, or the combined 18-24 years, 25-34, 35-44, 45-54, 55-64 years vs. >65 years) is about 0.685 times or 0.766 times, respectively, compared to those not in possession of cocaine or crack with the same level of biological sex, marital status, education, possession of firearm and employment status. The respective 95% confidence interval of the odds ratio for cocaine and crack are OR: 0.685; 95% CI: 0.602-0.779; p<0.0001 and OR: 0.766; 95% CI: 0.631-0.929; p=0.0068, respectively. Additionally, the possession of heroin and ecstasy at the time of arrest were statistically significant at the same level of biological sex, marital status, education, possession of firearm, and employment status. The 95% confidence interval of the odds ratio for heroin and ecstasy were OR: 1.431; 95% CI: 1.213- 1.689; p<0.0001 and OR: 2.659; 95% CI: 1.212- 5.831; p=0.0147, respectively. Notably, there was no statistically significant relationship with possession of phencyclidine, methamphetamines, prescription drugs, or any other psychoactive substance (p>0.05). See results of logistic regression analysis in Table 5.

**Table 5:** Odds of age at arrest by possession of substance at the time of arrest controlling for sociodemographic attributes in a logistic regression

Characteristics	Logistic Regression of age and possession of substance		
	Adjusted odds ratio	95% confidence Interval	p-value
Marijuana			
Yes	1.362	1.159-1.602	0.0002
No			
Cocaine			
Yes	0.685	0.602-0.779	<0.0001
No			
Crack			
Yes	0.766	0.631-0.929	0.0068
No			
Opioid			
Yes	1.431	1.213-1.689	<0.0001
No			
Phencyclidine (PCP)			
Yes			
No	1.011	0.327-3.119	0.9853
MDMA (ecstasy)			
Yes	2.659	1.212-5.831	0.0147
No			
Methamphetamines			
Yes	1.067	0.941-1.210	0.3092
No			
Prescription opioids			
Yes	1.095	0.842-1.424	0.4966
No			
Other drugs			
Yes	0.885	0.609-1.285	0.52
No			

## Discussion

Our study showed that the majority of inmates fall within the age groups 25-34 years and 35-44 years, with 31.50% and 27.98%, respectively. These age groups have been documented in the literature to have the highest criminal offenses rates [21,22]. The racial distribution was dominated by non-Hispanic White (NHW) and non-Hispanic Black populations, in keeping with trends reported by the Federal Bureau of Prisons, in which 70% of inmates are non-Hispanics. Our population sample was predominantly male, with three out of four (75%) inmates being male. This finding is somewhat consistent with the Federal Bureau of Prisons statistics showing that 93% of all inmates in the United States are males. The proportion of inmates reduced with the higher educational level of inmates.

Drug offenses are most common in the United States, representing 46.1% of all criminal offenses [22]. The rate of drug offenses is more than double that of weapons, explosives, and arson (the second most common offense with a prevalence of 20.7%). A major limitation of our study is the lack of data on alcohol use. Many studies have consistently reported alcohol amongst substances used by the inmate population. In a systematic review and meta‐regression analysis in recently incarcerated men and women, Fazel et al. found that alcohol use disorder was highly prevalent in prisoners, with a pooled estimate of 24% (95% CI: 21-27). They also reported that substance use disorder was as high as the alcohol estimates and possibly higher in female prisoners, with a pooled estimate of 51% (95% CI: 43-58) [23].

Regarding substance use among inmates in low- and middle-income countries, Mundt et al. found a pooled prevalence of 16% for alcohol use during imprisonment, with wide variations in geographical prevalence [24]. However, the lifetime prevalence of alcohol use among inmates in the Eldoret prison in Western Kenya was 65.1% [5]. Using the WHO-ASSIST (Alcohol, Smoking, and Substance Involvement Screening Test), Holmwood and collaborators reported that in male inmates in South Australia, the six most common substances used at high and moderate risk levels were tobacco, cannabis, amphetamines, opiates, alcohol, and sedatives [25]. The other was slightly different in women, with tobacco first and alcohol last. In a study of 801 female inmates incarcerated in the Minnesota Department of Corrections state prison system, Proctor reported that 70.0% were dependent on at least one substance, and 7.9% met the criteria for substance abuse [6].

We found that inmates with a history of stimulant or hypnotic use were most likely to have another psychoactive substance during the time of their arrest. The logistic regression models show that multiple sociodemographic factors are associated with the possession of substances at the time of the arrest. These factors have also been associated with a higher risk of criminal recidivism [9]. Considering the odds of substance possession at the time of arrest by sociodemographic attributes, they appear to be associated with multiple substance use. Hakansson and Berglund reported a significant association between polysubstance use at baseline and criminal recidivism, with an increase in the risk of recidivism for a higher number of substances used prior to incarceration [9].

Thus, this suggests that for inmates in the United States, substance use and the possession of substances at the time of arrest have multifactorial risk factors. Hence, in order to address these factors, the focus needs to be directed not only towards treatment referrals, evidence-based addiction treatment, and structured follow-up of prisoners' substance use problems but also on addressing underlying social determinants of health.

The strengths of our study are its huge sample size and depth of data that was collected through the survey. The major limitations of the study include the lack of cause-and-effect relationship and possible recall bias inherent to all surveys. However, this study sheds more light on the problem of substance use in incarcerated populations.

## Conclusions

Overall, there is a high rate of imprisonment in the US. This rate of imprisonment due to drug-related crimes has been rising over the last decade. There have been studies correlating imprisonment with drug-related criminal behavior; a significant correlation exists with substance use and criminality, leading to imprisonment. With a better understanding of correlated factors, we can create pathways to prevent recurrence by providing the appropriate mental health resources to prisoners. Reviewing factors associated with SUD and criminality, we can attempt to review pathways that can aid in creating targeted interventions for the most vulnerable groups, notably creating a pathway towards a stronger and more accessible mental health program while in prison and after release. 
